# Supplementary material for: AdRoit is an accurate and robust method to infer complex transcriptome composition
Source: Commun Biol. 2021 Oct 22;4:1218. doi: 10.1038/s42003-021-02739-1 (PMC8536787; doi:10.1038/s42003-021-02739-1)
Supplement: Supplementary file 2 — Supplementary Information [file 42003_2021_2739_MOESM2_ESM.pdf]

Supplementary information

AdRoit is an accurate and robust method to infer complex transcriptome composition

Tao Yang[1], Nicole Alessandri-Haber[1], Wen Fury[1], Michael Schaner[1], Robert Breese[1], Michael LaCroix-Fralish[2],

Jinrang Kim[1], Christina Adler[1], Lynn E. Macdonald[1], Gurinder S. Atwal[1], Yu Bai[1, *]

Affiliations

1. Regeneron Pharmaceuticals, Inc., Tarrytown NY 10591
2. Cellular Longevity, Inc., San Francisco, CA 94103

*Corresponding author

Supplementary Figures

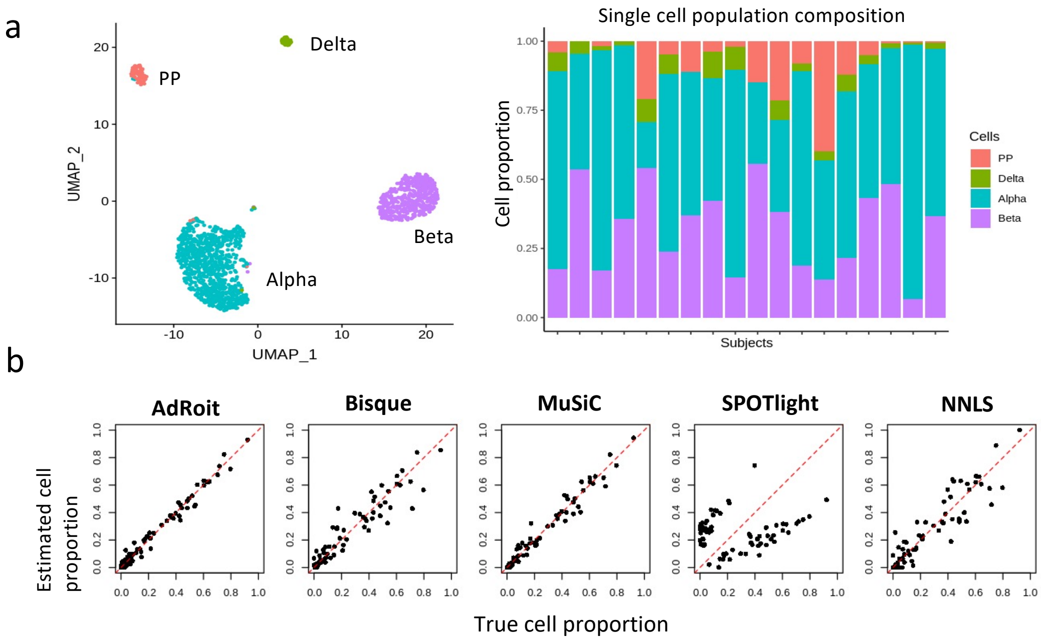

c

	AdRoit	Bisque	MuSiC	SPOTlight	NNLS
**mAD**	0.026	0.056	0.031	0.24	0.066
**RMSD**	0.036	0.082	0.046	0.25	0.095
**Pearson**	0.99	0.94	0.98	0.16	0.93
**Spearman**	0.97	0.94	0.98	0.06	0.91

Supplementary Figure 1. Benchmark five methods on human pancreatic islets data. a, Human islets single cell data contains 4 cell types (Alpha, Beta, PP, and Delta cells)[1] from 18 subjects.The cell proportion varies across different subjects. b, c, AdRoit achieves a leading accuracy when applied to the bulk data synthesized from the single cell data. Each dot on the scatterplot is a cell type from one subject. The reference and gene weight estimations used for deconvoluting each synthetic bulk sample exclude the data of that sample (i.e., leave-one-out).

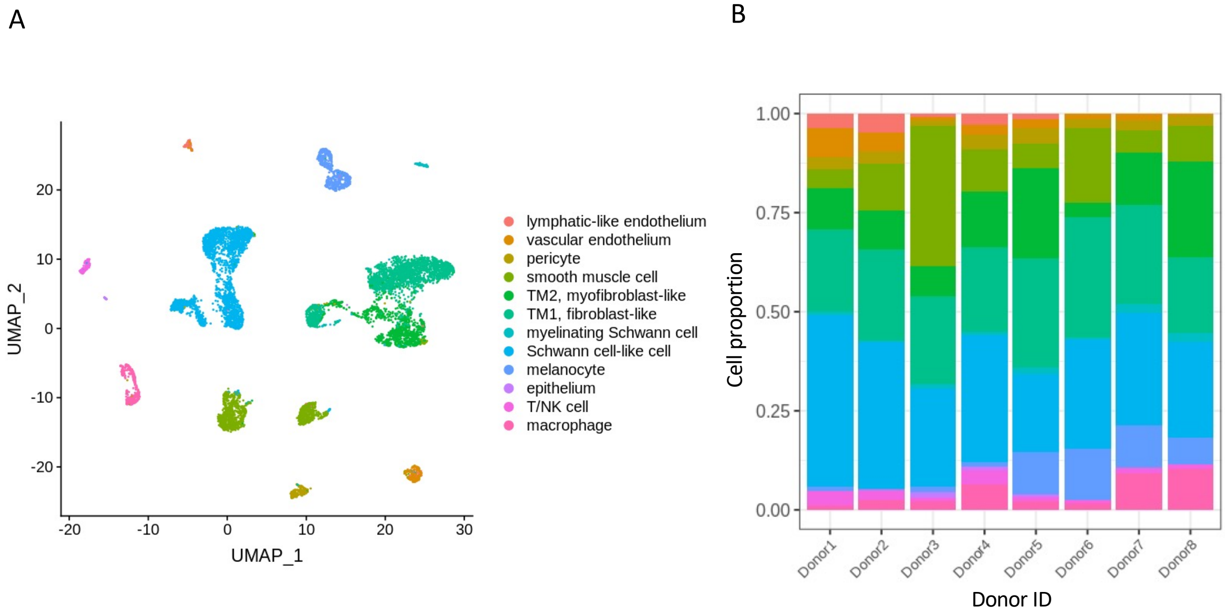

Supplementary Figure 2. Trabecular meshwork single cell data reveals 12 cell types from 8 donors[2]**. a,** UMAP[3] projection of single cells shows 12 distinct cell types. **b,** The cell proportion of each cell type varies across different donors.

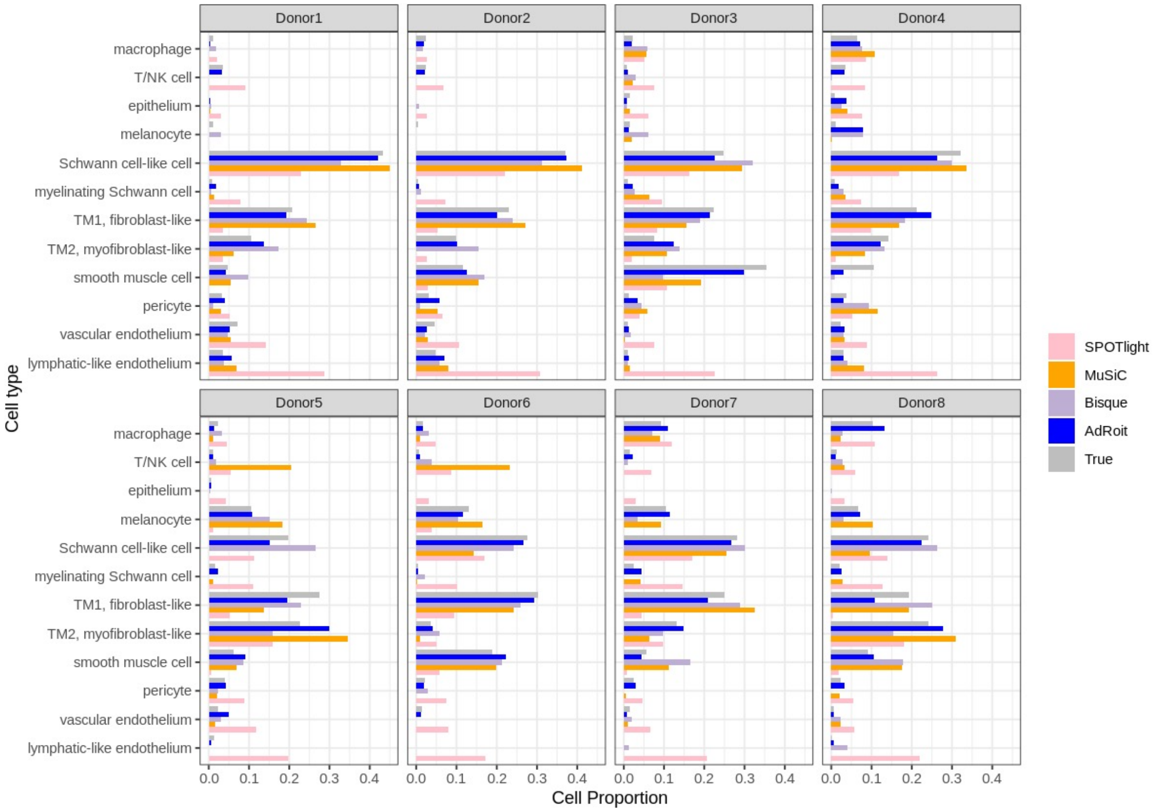

Supplementary Figure 3. Comparison of estimated cell type proportions by AdRoit, Bisque[4], MuSiC[5] and SPOTlight[6] on the trabecular meshwork data[2] of each donor. For each sample, AdRoit's estimates (blue bars) are more consistent with the true cell type proportions (grey bars) than the other methods.

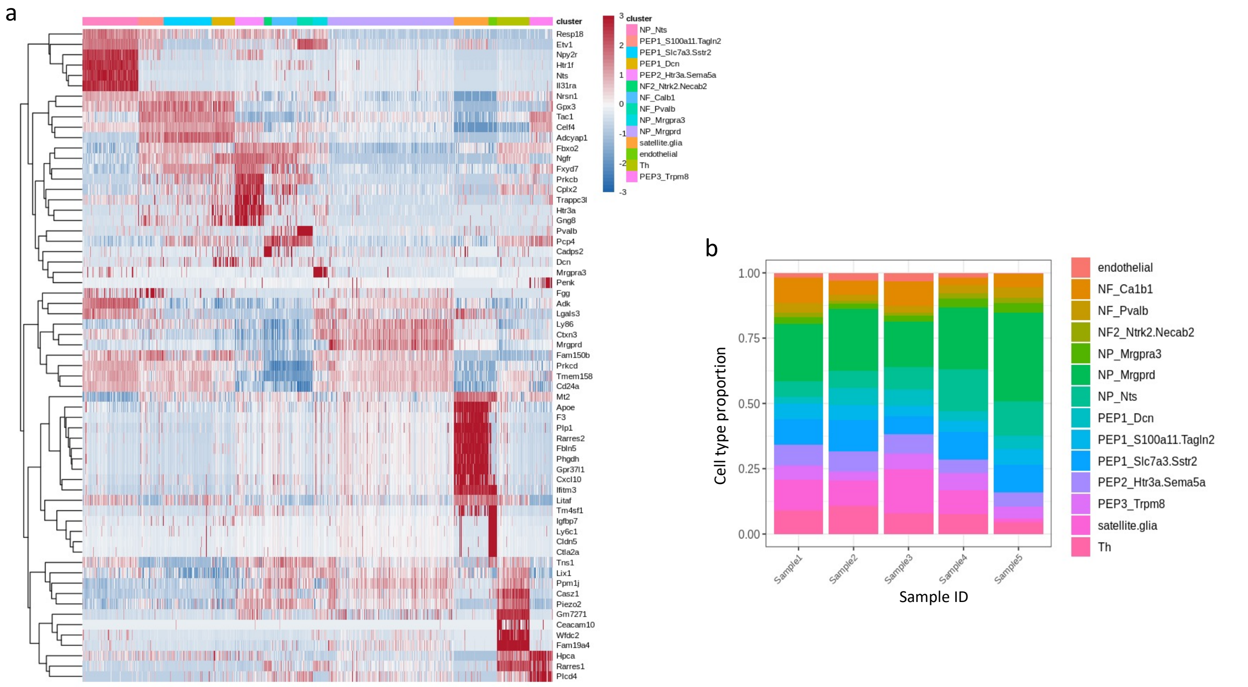

Supplementary Figure 4. Dorsal root ganglion single cell shows 14 cell types including 3 subtypes of neurofilament-containing neurons, 3 subtypes of non-peptidergic neurons, and 5 subtypes of peptidergic neurons. a, Heatmap of top markers shows the distinction and the similarity between the cell subtypes. b, The proportion of each cell type varies from 0.5% to 33.71% across different samples.

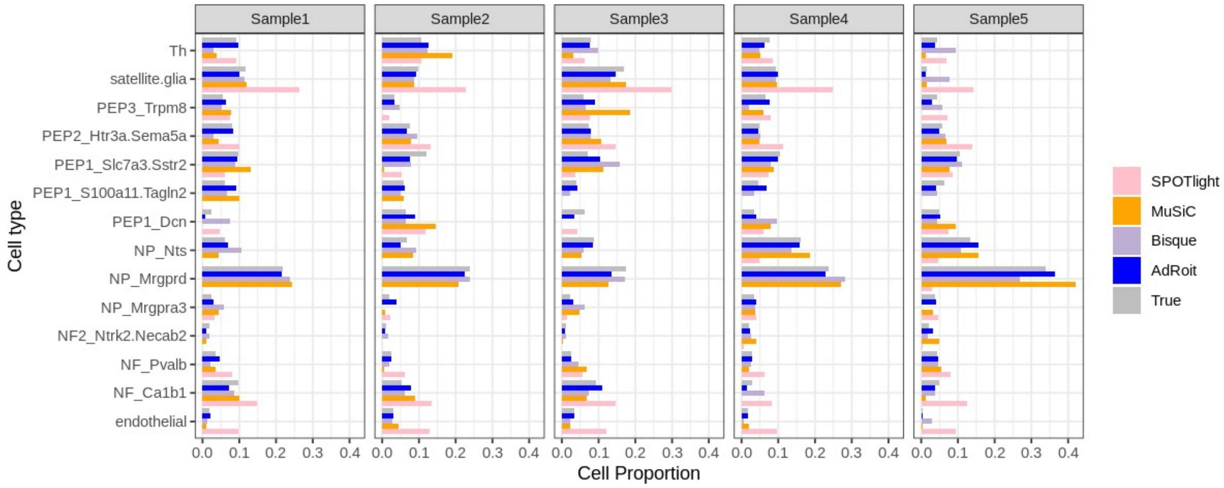

Supplementary Figure 5. Comparison of estimated cell type proportions by AdRoit, Bisque[4], MuSiC[5] and SPOTlight[6] on the synthetic dorsal root ganglion bulk samples. For each sample, AdRoit's estimates (blue bars) are more consistent with the true cell type proportions (grey bars) than the other methods.

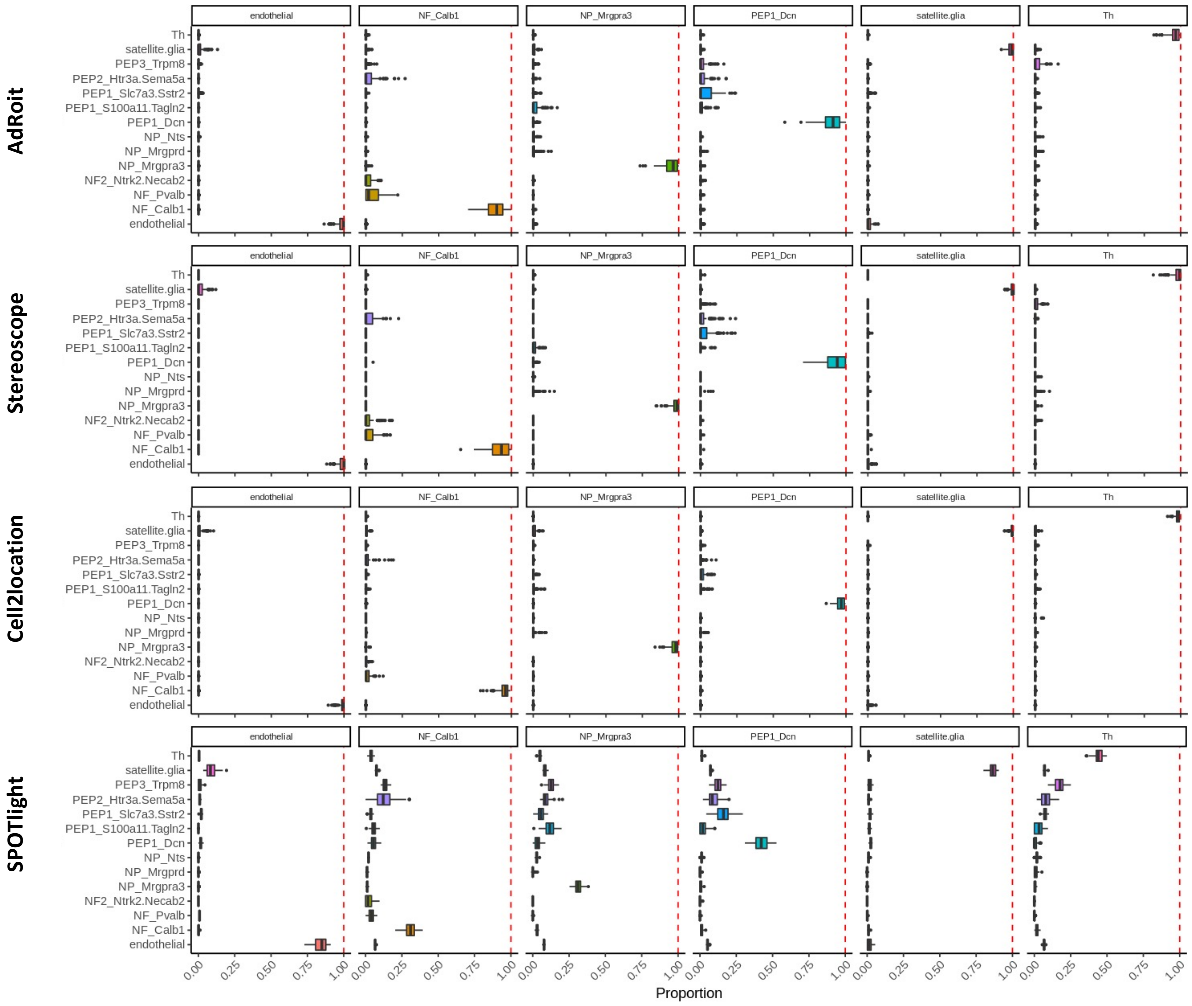

Supplementary Figure 6. Comparing the deconvolution performance on simulated spatial spots containing a pure cell population. All methods except SPOTlight accurately estimate the present cell type close to 1 and the absent cell types near 0. Simulations were done by sampling cells from a given cell type and adding up the UMI counts per gene. For each of the 14 DRG cell types, we repeated the simulation 100 times. The results of 6 cell types are shown here. The complete estimations for all 14 cell types are listed in Supplementary Table 11. Bars of the boxplots indicate the interquartile ranges.

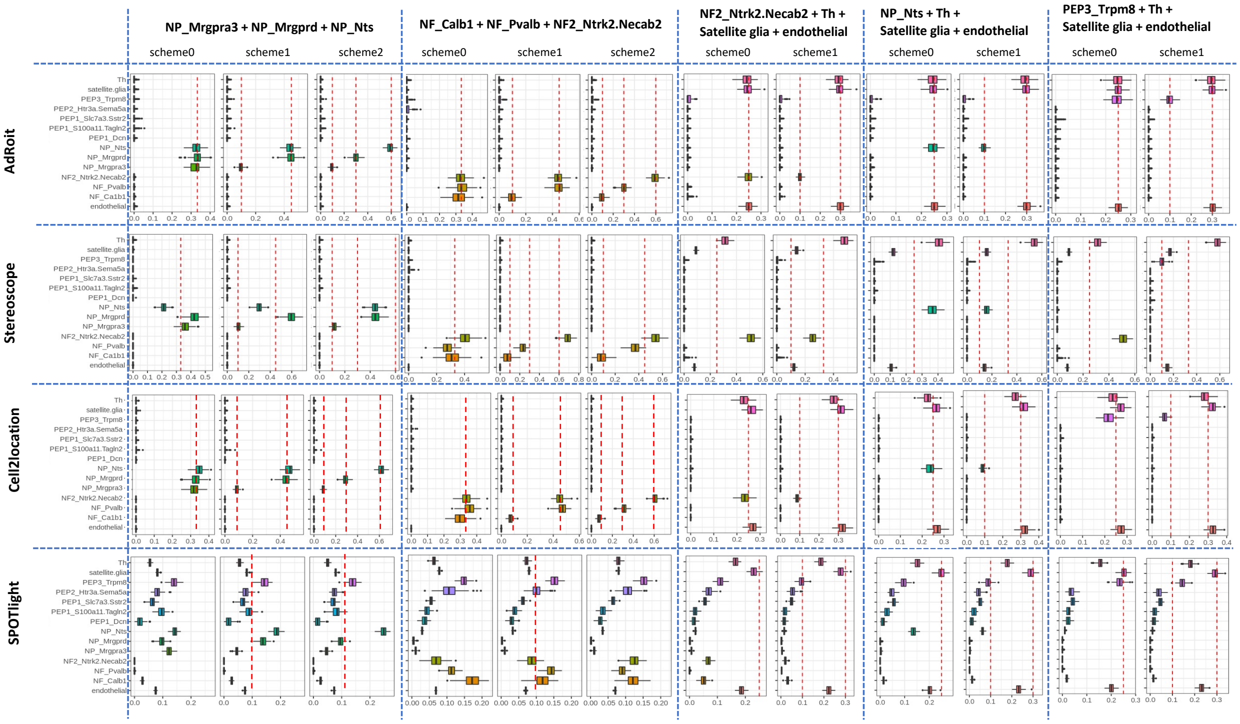

Supplementary Figure 7. Compare deconvolution algorithms on the simulated spatial spots using additional cell mixing schemes. 5 more sets of mixed spatial spots were simulated: 1) mixture of 3 neurofilament-containing neuron subtypes (NF); 2) mixture of 3 non-peptidergic (NP) neuron subtypes; 3) NF2_Ntrk2.Necab2 with Th, satellite glia and endothelial; 4) NP_Nts with Th, satellite glia and endothelial; and 5) PEP3_Trpm8 with Th, satellite glia and endothelial. Each simulation was repeated 100 times. Consistently for all simulation schemes, AdRoit's estimates are closer to the ground truth proportions (red lines), whereas the other methods show more deviations. Bars of the boxplots indicate the interquartile ranges.

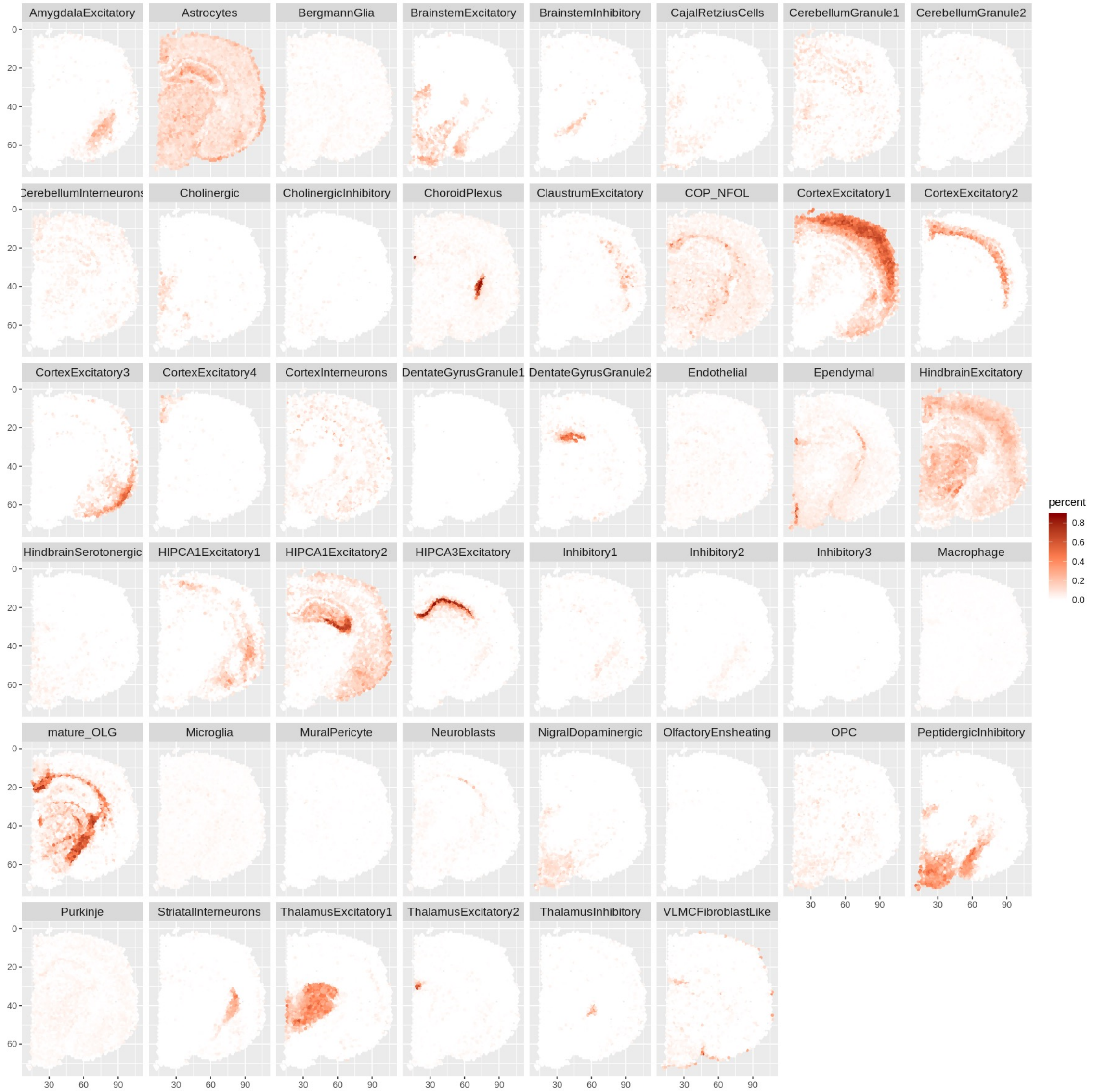

Supplementary Figure 8. Spatial mapping of 46 brain cell types by AdRoit quantitatively depicts the content in each spot. Spatial transcriptomics data was downloaded from 10x Genomics (see Data availability in the main text). The single cell reference data was sampled from a published mouse brain atlas[7] and consolidated into 46 cell types (see Methods in the main text).

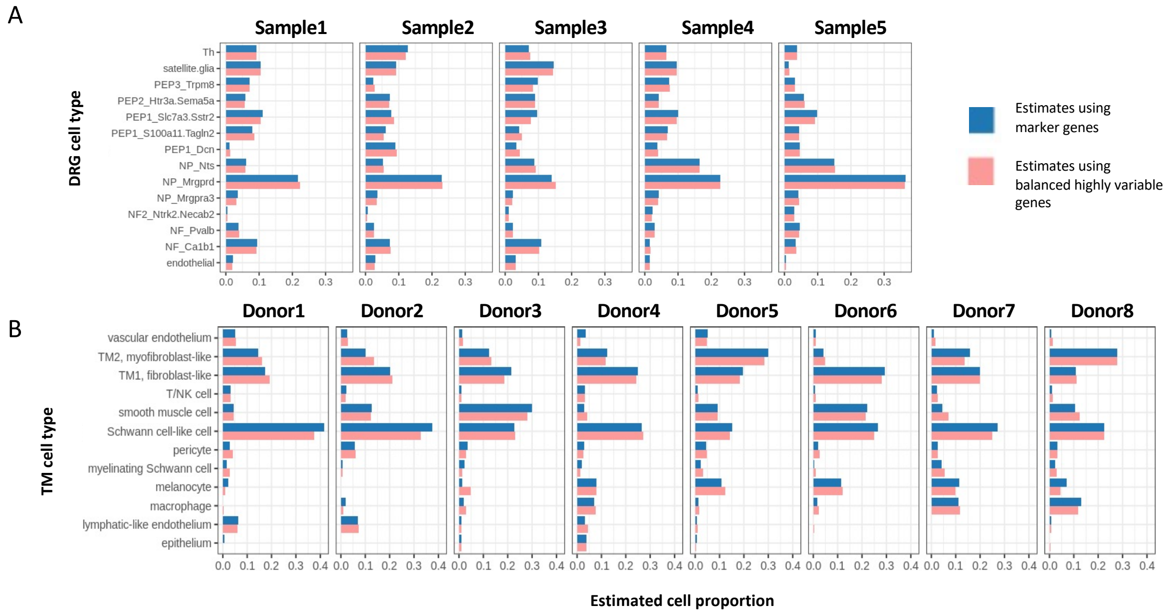

Supplementary Figure 9. Estimation of cell proportions using either marker genes or balanced highly variable genes yields comparable results in dorsal root ganglion (DRG) data (a) and trabecular meshwork (TM) data (b). The union of the top 200 markers (based on fold change) per cell type is compared with the top 2000 balanced highly variable genes.

Supplementary References

1. Xin, Y. *et al.* RNA Sequencing of Single Human Islet Cells Reveals Type 2 Diabetes Genes. *Cell Metab.* (2016) doi:10.1016/j.cmet.2016.08.018.
2. Patel, G. *et al.* Molecular taxonomy of human ocular outflow tissues defined by single-cell transcriptomics. *Proc. Natl. Acad. Sci.* **117**, 12856 LP – 12867 (2020).
3. McInnes, L., Healy, J., Saul, N. & Großberger, L. UMAP: Uniform Manifold Approximation and Projection. *J. Open Source Softw.* (2018) doi:10.21105/joss.00861.
4. Jew, B. *et al.* Accurate estimation of cell composition in bulk expression through robust integration of single-cell information. *Nat. Commun.* (2020) doi:10.1038/s41467-020-15816-6.
5. Wang, X., Park, J., Susztak, K., Zhang, N. R. & Li, M. Bulk tissue cell type deconvolution with multi-subject single-cell expression reference. *Nat. Commun.* (2019) doi:10.1038/s41467-018-08023-x.
6. Elosua-Bayes, M., Nieto, P., Mereu, E., Gut, I. & Heyn, H. SPOTlight: seeded NMF regression to deconvolute spatial transcriptomics spots with single-cell transcriptomes. *Nucleic Acids Res.* (2021) doi:10.1093/nar/gkab043.
7. Zeisel, A. *et al.* Molecular Architecture of the Mouse Nervous System. *Cell* (2018) doi:10.1016/j.cell.2018.06.021.
8. Xin, Y. *et al.* Pseudotime ordering of single human B-cells reveals states of insulin production and unfolded protein response. *Diabetes* (2018) doi:10.2337/db18-0365.

9. Gutierrez, G. D. *et al.* Gene signature of proliferating human pancreatic a cells. *Endocrinology* (2018) doi:10.1210/en.2018-00469.
