# Supplementary material for: AdRoit is an accurate and robust method to infer complex transcriptome composition
Source: Commun Biol. 2021 Oct 22;4:1218. doi: 10.1038/s42003-021-02739-1 (PMC8536787; doi:10.1038/s42003-021-02739-1)
Supplement: Supplementary file 3 — Description of Additional Supplementary Files [file 42003_2021_2739_MOESM3_ESM.pdf]

Description of Additional Supplementary Files

File name: Supplementary Data 1

Description: Cell counts and proportions in the human pancreatic islets single cell dataset and the accompanying deconvolution results.

File name: Supplementary Data 2

Description: Cell counts and proportions in the human trabecular meshwork single cell dataset and the accompanying deconvolution results.

File name: Supplementary Data 3

Description: Estimates of cell proportions in the mixtures of myeloid and lymphoid cell types by AdRoit.

File name: Supplementary Data 4

Description: True and estimated cell proportions in the bulk RNA-seq data synthesized using 6 out of the 12 human trabecular meshwork cell types.

File name: Supplementary Data 5

Description: Sampled mouse brain single cells from Zeisel et al and the consolidated cell type annotation

File name: Supplementary Data 6

Description: True and estimated cell proportions in the simulated bulk samples of 30 randomly selected mouse brain cell types.

File name: Supplementary Data 7

Description: True and estimated cell proportions in the synthetic bulk RNA-seq data of mouse dorsal root ganglion

File name: Supplementary Data 8

Description: Estimated cell proportions of 2900 spatial transcriptome spots simulated by sampling and pooling multiple cell types from the mouse dorsal root ganglion single cell data.

File name: Supplementary Data 9

Description: Estimated cell percentages of 6000 spatial transcriptome spots simulated to evaluate the accuracy and sensitivity in deconvoluting rare cell types

File name: Supplementary Data 10

Description: Read count per gene from the 70 real bulk RNA-seq samples of human pancreatic islets, together with the donor information, the available HbA1C and RNA-FISH measurements.

File name: Supplementary Data 11

Description: AdRoit-estimated cell proportions in the 70 real bulk RNA-seq samples of human pancreatic islets.

File name: Supplementary Data 12

Description: AdRoit-estimated cell proportions in the real mouse brain spatial transcriptome spots.
