# Supplementary material for: AdRoit is an accurate and robust method to infer complex transcriptome composition
Source: Commun Biol. 2021 Oct 22;4:1218. doi: 10.1038/s42003-021-02739-1 (PMC8536787; doi:10.1038/s42003-021-02739-1)
Supplement: Supplementary file 16 — Reporting Summary [file 42003_2021_2739_MOESM16_ESM.pdf]

nature research

Corresponding author(s): Yu Bai

Last updated by author(s): Sep 9, 2021

Reporting Summary

Nature Research wishes to improve the reproducibility of the work that we publish. This form provides structure for consistency and transparency in reporting. For further information on Nature Research policies, see our Editorial Policies and the Editorial Policy Checklist.

Statistics

For all statistical analyses, confirm that the following items are present in the figure legend, table legend, main text, or Methods section.

n/a	Confirmed	
☐	☒	The exact sample size (*n*) for each experimental group/condition, given as a discrete number and unit of measurement
☐	☒	A statement on whether measurements were taken from distinct samples or whether the same sample was measured repeatedly
☐	☒	The statistical test(s) used AND whether they are one- or two-sided<br>*Only common tests should be described solely by name; describe more complex techniques in the Methods section.*
☒	☐	A description of all covariates tested
☒	☐	A description of any assumptions or corrections, such as tests of normality and adjustment for multiple comparisons
☐	☒	A full description of the statistical parameters including central tendency (e.g. means) or other basic estimates (e.g. regression coefficient) AND variation (e.g. standard deviation) or associated estimates of uncertainty (e.g. confidence intervals)
☒	☐	For null hypothesis testing, the test statistic (e.g. *F*, *t*, *r*) with confidence intervals, effect sizes, degrees of freedom and *P* value noted<br>*Give P values as exact values whenever suitable.*
☒	☐	For Bayesian analysis, information on the choice of priors and Markov chain Monte Carlo settings
☒	☐	For hierarchical and complex designs, identification of the appropriate level for tests and full reporting of outcomes
☐	☒	Estimates of effect sizes (e.g. Cohen's *d*, Pearson's *r*), indicating how they were calculated

Our web collection on statistics for biologists contains articles on many of the points above.

Software and code

Policy information about availability of computer code

Data collection	Sample de-multiplexing, alignment, filtering, and UMI counting were conducted using Cell Ranger Single-Cell Software Suite (10X Genomics, v2.0.0). Mouse mm10 Genome assembly and UCSC gene model were used for the alignment.
Data analysis	The statistical analyses were done with R statistical software (v3.6.0) and python (v3.7.2). The packages used include Seurat (v3.0.1), scanpy (v1.6.0), dplyr (v0.8.0.1), doParallel (v1.0.14), data.table (v1.12.4), fitdistrplus (v1.1-1), nnls (v1.4), MuSiC (v0.1.1), BisqueRNA (v1.0.4), SPOTlight (v1.0.4), Cell2location (v0.05-alpha), Stereoscope(v_03). The software developed in this manuscript was deposited at Github (https://github.com/TaoYang-dev/AdRoit).

For manuscripts utilizing custom algorithms or software that are central to the research but not yet described in published literature, software must be made available to editors and reviewers. We strongly encourage code deposition in a community repository (e.g. GitHub). See the Nature Research guidelines for submitting code & software for further information.

Data

Policy information about availability of data

All manuscripts must include a data availability statement. This statement should provide the following information, where applicable:

- Accession codes, unique identifiers, or web links for publicly available datasets
- A list of figures that have associated raw data
- A description of any restrictions on data availability

The mouse DRG single cell data were deposited at NCBI GEO (accession number: GSE163252). The bulk RNA-Seq and RNA-FISH data for human pancreatic islets were initially published as aggregated data where the data processing and experimental procedure were described therein. We acquired the individual sample data from the authors and released them along with the current study (Supplementary Data 10). The other public data analyzed in this study were obtained using GEO accession number GSE81608 (human pancreatic islets single cell data), NCBI SRA accession number PRJNA616025 (human trabecular meshwork single cell data),

and NCBI SRA accession number SRP135960 (mouse brain single cell data). The 10x Genomics PBMC data and the Visium mouse brain spatial transcriptomics data were downloaded from 10x Genomics website (https://support.10xgenomics.com/single-cell-gene-expression/datasets/1.1.0/pbmc3k, https://support.10xgenomics.com/spatial-gene-expression/datasets/1.1.0/V1_Adult_Mouse_Brain).

Field-specific reporting

Please select the one below that is the best fit for your research. If you are not sure, read the appropriate sections before making your selection.

- [x] Life sciences
- [ ] Behavioural & social sciences
- [ ] Ecological, evolutionary & environmental sciences

For a reference copy of the document with all sections, see nature.com/documents/nr-reporting-summary-flat.pdf

Life sciences study design

All studies must disclose on these points even when the disclosure is negative.

Sample size	Single cell RNA-sequencing was performed on dorsal root ganglion tissue of 5 mice. The goal of doing this experiment is to characterize the cell population. Five mice would generate sufficient number of neuronal cells that reflect the cell population.
Data exclusions	No data excluded.
Replication	Single cell RNA-sequencing was performed on 5 mice. The computational method described in this paper was applied to each of them to ensure the reproducibility of estimation.
Randomization	Randomization is not relevant. There is only one wild type group. The goal is not to compare between groups.
Blinding	Not relevant. No group comparison was done.

Reporting for specific materials, systems and methods

We require information from authors about some types of materials, experimental systems and methods used in many studies. Here, indicate whether each material, system or method listed is relevant to your study. If you are not sure if a list item applies to your research, read the appropriate section before selecting a response.

Materials & experimental systems

n/a	Involved in the study	
x		Antibodies
x		Eukaryotic cell lines
x		Palaeontology and archaeology
	x	Animals and other organisms
x		Human research participants
x		Clinical data
x		Dual use research of concern

Methods

n/a	Involved in the study	
x		ChIP-seq
x		Flow cytometry
x		MRI-based neuroimaging

Animals and other organisms

Policy information about studies involving animals; ARRIVE guidelines recommended for reporting animal research

Laboratory animals	The five mice used were C57BL/6 mice. All of them were 18 weeks old. Three of the five are male, the other two are female.
Wild animals	The study did not involve wild animals.
Field-collected samples	The study did not involve samples collected from the field.
Ethics oversight	Research involving the use of live animals were approved by the Regeneron Institutional Animal Care and Use Committee (IACUC).

Note that full information on the approval of the study protocol must also be provided in the manuscript.
